# The Time-Dependent Interfacial Adhesion between Artificial Rock and Fresh Mortar Modified by Nanoclay

**DOI:** 10.3390/nano14090776

**Published:** 2024-04-30

**Authors:** Xiaoyun Wang, Kim Van Tittelboom, Jiaolong Zhang, Yaxin Tao, Yao Rong, Luc Taerwe, Geert De Schutter, Yong Yuan

**Affiliations:** 1 College of Civil Engineering, Tongji University, Shanghai 200092, China; xiaoyun.wang@ugent.be (X.W.);; 2 Magnel-Vandepitte Laboratory for Structural Engineering and Building Materials, Ghent University, 9052 Ghent, Belgium; 3 Belgium-China Joint Laboratory for Industrialized Construction, Shanghai 200092, China; 4 Institute of Building Materials, ETH Zurich, 8092 Zurich, Switzerland; 5 Jiangxi Transportation Institute Co., Ltd., Nanchang 330200, China; 6 State Key Laboratory of Disaster Reduction in Civil Engineering, Tongji University, Shanghai 200092, China

**Keywords:** interface, adhesive property, 3D concrete printing, mountain tunnel lining

## Abstract

The time-dependent interfacial adhesion between rock and fresh mortar is key for printing concrete linings in mountain tunnels. However, a scientific deficit exists in the time-dependent evolution of the interfacial adhesion, which can cause adhesion failure when printing tunnel lining. Nanoclay has the potential to increase the interfacial adhesion and eliminate the adhesion failure. Before the actual printing of tunnel linings, the time-dependent interfacial adhesion between artificial rock and fresh mortar modified by nanoclay should be understood. This paper studied the time-dependent interfacial adhesion based on fast tack tests, fast shear tests, and isothermal calorimetry tests. With the addition of nanoclay, the maximum tensile stress and the maximum shear stress increased. Compared with a reference series, the maximum interfacial tensile stress in a 0.3% nanoclay series increased by 106% (resting time 1 min) and increased by 209% (resting time 32 min). A two-stage evolution of the interfacial adhesion was found with the addition of nanoclay. In the first stage, the time-dependent interfacial adhesion increased rapidly. A 0.3% NC series showed an increase rate six times higher than that of the reference series. As the matrices aged, the increase rate slowed down and followed a linear pattern of increase, still higher than that of the reference series. The stiffening of fresh matrices resulted in the interface failure mode transition from a ductile failure to a brittle failure. The effect of nanoclay on flocculation and on accelerating the hydration contributed to the time-dependent interfacial adhesion between artificial rock and fresh mortar.

## 1. Introduction

3D printed concrete [1] has the potential to replace shotcrete [2] to construct linings in hard rock mountain tunnels, with the merits of a high degree of automation and rebound elimination [3]. While printing, interfacial adhesion forms between the freshly printed concrete and the rock. The interfacial adhesion, which is crucial for printing tunnel linings [4], is characterized by the external loads that must be applied to separate the two components [5], e.g., force and torque. In lab-scale tunnel lining printing, interfacial adhesion failures between fresh concrete and substrate were observed [6]. This highlighted the necessity of investigating and improving the interfacial adhesion. Tensile interfacial failure and shear interfacial failure were two typical failure modes found during the lab-scale tunnel lining printing [7]. This stressed the importance of studying interfacial adhesion when subjecting 3D printed concrete to tensile stresses or shear stresses.

**Figure 1:**
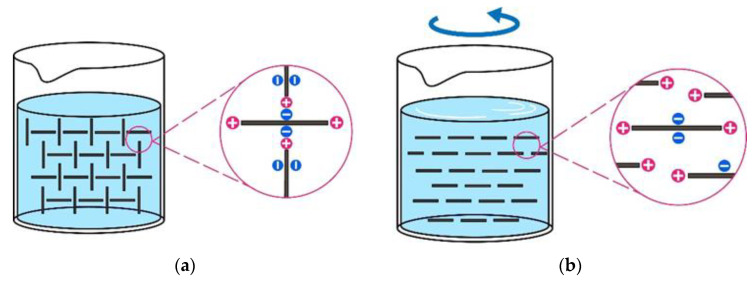
Illustration of the internal microstructure of a suspension modified by nanoclay: (**a**) the internal microstructure of nanoclay when the matrix is at rest; (**b**) the internal microstructure of nanoclay when the matrix is subject to shearing.

The mix proportions [8,9] and the age of the cementitious material [10] can significantly influence the interfacial adhesion. The addition of admixtures is effective in modifying the interface adhesion of fresh cementitious materials [11]. Nanomaterials, e.g., nanoclay, have been used to increase the buildability of 3D printed concrete [12,13]. Nanoclay has opposite charges on the surfaces of particles compared to the edges [14]. As shown in Figure 1, the black lines represent the rod-shaped particles of nanoclay, which is commonly used in 3D concrete printing [15]. The particles are positively charged on the ends and negatively charged along the axis. When the cementitious material is at rest, the edge-to-surface interaction results in the formation of a microstructural network in the material, as shown in Figure 1a. The microstructural network generates higher viscosity. When the matrix is subjected to shear, the network dissociates, resulting in a decreased viscosity, as shown in Figure 1b. The addition of nanoclay also contributes to the structural build-up of cementitious materials and leads to an instant increase in the yield stress in the fresh state [16,17,18]. Furthermore, the addition of nanoclay enhances the adhesive properties of fresh cementitious materials. For instance, Kawashima et al. [19] conducted tack tests on fresh cement pastes and found that nanoclay increased the peak force required for debonding.

Considering the mentioned effects of nanoclay on cementitious materials, adding nanoclay in printable concrete can be beneficial for printing tunnel linings. However, one issue must be investigated before actually using nanoclay in printable materials, i.e., the influence of nanoclay on the time-dependent interfacial adhesion between rock and fresh cementitious materials. Previous research has emphasized the effect of the age of printable concrete on the interface bond shear strength [3,20]. The first challenge encountered in this issue is to find applicable test methods to study the time-dependent interfacial adhesion. In a previous study, the tack test was recommended by Kawashima et al. [19] as a suitable method to study the adhesive properties of fresh cement paste. The test recorded the tensile force required to separate the interface between fresh cementitious materials and substrates [21,22]. Tao et al. [7] further compared the test results of the tack test and a modified shear test with lab-scale tunnel lining printing. The comparison confirmed that the aforementioned test methods can be used to predict the adhesive properties of fresh cementitious materials for tunnel lining printing.

However, the tests show a deficiency, especially when studying the interfacial adhesion of fresh cementitious materials modified by nanoclay. The structural build-up of cementitious materials modified by nanoclay is time-dependent. The material properties evolve significantly in the minutes after the stop of the flow [17,23,24]. This indicates the interfacial adhesion can also evolve quickly, within minutes, after the formation of the interface. The tack test and the modified shear test normally require more than 5 min to form the interface. In the time elapsed during the interface formation, a significant evolution of the interfacial adhesion may happen, thus rendering those tests inappropriate. Additionally, there is little research focused on the time-dependent interfacial adhesion between rock and fresh mortar modified by nanoclay. Further research on this point is needed to reveal the scientific nature of the time-dependent interfacial adhesion and to provide practical insights for printing in situ tunnel lining.

This paper studies the effect of nanoclay on the time-dependent interfacial adhesion between artificial rock and fresh mortar. Two fast tests were developed, namely a fast tack test and a fast shear test, capable of forming the interface in 15 s after the end of mixing. Four mortar matrices with the dosage of nanoclay ranging from 0% to 0.3% were tested using the modified tests. The time-dependent interfacial adhesion was tested from the 1st minute until the 32nd minute after the formation of the interface. Meanwhile, isothermal calorimetry tests were conducted to monitor the hydration process of the fresh matrices.

## 2. Materials and Methods

### 2.1. Materials and Mixture Proportions

Four mixture proportions were used in this study. In all mixtures, the water-to-cement ratio was fixed at 0.35 and the cement-to-sand ratio was fixed at 1. One reference mixture (REF) and three mixtures with different dosages of nanoclay (NC) are shown in Table 1. The selection of the mixture proportions is based on the mixture used in the lab-scale 3D printing concrete at the Belgium–China Joint Laboratory for Industrialized Construction [6]. The low water-to-cement ratio and the high cement-to-sand ratio are beneficial for the buildability of the mortar [25].

The cement type used was CEM I 52.5 N, provided by the Belgian Holcim company (Brussels, Belgium),. The cement had a specific gravity of 3160 kg/m^3^ and a Blaine specific area of 408 m^2^/kg, with the chemical compositions shown in Table 2. CEN Standard sand was used, provided by the German company, Normensand GmbH (Muenster, Germany). The specific grain size distribution of the sand ranged between 0.08 mm and 2.00 mm. Tap water was used to prepare the mixtures [6].

**Figure 2:**
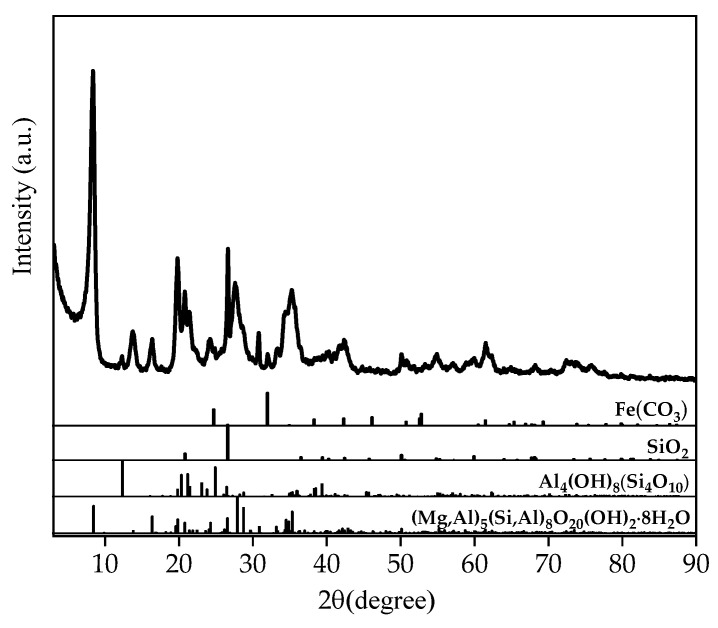
The XRD test result of the nanoclay.

The nanoclay used was a commercially available product provided by Faber&VanderEnde BV (Almere, Netherlands), Acti-gel^®^, that is rich in palygorskite. The Acti-gel is made from a wet process that significantly removes most of the grit (SiO_2_ and CaCO_3_) and other impurities (Smectite). Palygorskite is a mineralogical material that is gaining increasing interest in modern construction [26]. The nanoclay had an average particle size of 1.5 to 2 microns in length and an average diameter of 30 angstroms. The chemical composition of the nanoclay is shown in Table 2. The X-ray Diffraction (XRD) test results of the nanoclay are shown in Figure 2. The main component of the nanoclay was palygorskite with the molecular formula of ${\left(\mathrm{Mg},\;\mathrm{Al}\right)}_{5}{\left(\mathrm{Si},\;\mathrm{Al}\right)}_{8}O_{20}\cdot {8H}_{2}O$.

In this study, ultra-high-performance concrete (UHPC) was used as the substrate to simulate hard rock. The high compressive strength of UHPC, i.e., 149 MPa, resembles that of hard rock in mountain tunnels [27]. The mixture proportions of the UHPC are shown in Table 3 [28]. The porosity of UHPC is around 6% [29], which is similar to natural hard rock, e.g., basalt [30]. In the UHPC, the cement was CEM III/A 52.5 R, provided by Dyckerhoff Germany (Wiesbaden, Germany). The sand and Basalt were provided by the Belgian Holcim company (Brussels, Belgium). The silica fume was provided by the company Elkem (Liège, Belgium). The filler was provided by the company Franzefoss Minerals (Sandvika, Norway). The superplasticizer was provided by the SIKA company (Ghent, Belgium). 

### 2.2. Mixing Procedure

All mortar mixtures were mixed by a planetary mortar mixer. All ingredients were stored in a laboratory room with a temperature of (20 ± 2) °C and a relative humidity of (50 ± 5)%. The following protocol was adopted for mixing the four cementitious matrices: (1) manually mixing cement powder with sand for 30 s; (2) adding nanoclay powder into water and stirring with a glass rod for 30 s (not applicable for the REF); (3) adding water to the premixed cement with sand, mixing with a rotational speed of 140 rpm for 30 s; (4) mixing with a rotational speed of 285 rpm for 30 s; (5) halting for 90 s; (6) mixing with a rotational speed of 285 rpm for 60 s.

### 2.3. Test Methods

#### 2.3.1. Isothermal Calorimetry Tests

The isothermal calorimetry tests were conducted using a TAM Air isothermal calorimeter. The temperature in the calorimeter was kept at (20 ± 0.02) °C. Four mixture proportions of cement pastes were tested for 1 h. The test was repeated twice for each mixture proportion. The mixture proportions were based on Table 1, although no sand was added. Each sample contained 14 g cement paste. The samples were first mechanically stirred for 2 min, then were immediately added into the ampoules. The ampoules were sealed and placed into the calorimeter within 5 min after the contact of water and cement.

#### 2.3.2. Fast Tack Tests

The fast tack test (FTT) aims to measure the interfacial adhesion under tensile stress between artificial rock and cementitious materials at a fresh state. The test is capable of forming the interface as early as 15 s after premixing. The FTT complements the deficiency in the traditional tack test [6] by testing the interfacial adhesion in the first several minutes after mixing. The FTT is applicable to measure the adhesion of the cementitious material with the addition of nanoclay. By setting various resting times in the FTT, the time-dependent development of interfacial adhesion (tensile) can be obtained. The FTT can provide the complete time-dependent development of the interfacial adhesion immediately after mixing.

The FTT consisted of three main steps, i.e., pre-shearing of the matrix, fast formation of the interface, and debonding of the interface with a certain resting time of the matrix. The basic steps of the traditional tack test are clarified by Tao et al. [6] and briefly mentioned as follows: Firstly, a rheometer with a parallel-plate geometry was used. A concrete plate (diameter 50 mm) was glued to the top plate serving as the rock substrate. Sandpaper covered the bottom plate to avoid slippage. A cylindrical mold (diameter 50 mm), without a top and bottom plate, was placed on the sandpaper. Then, matrix was taken from a mixer bowl and filled into the mold. The bottom of the fresh sample adhered to the sandpaper. After levelling the top of the sample, the mold was gently removed. The rheometer was started, and the substrate was lowered to a certain position. During the process, the substrate and the sample contacted, forming the interface. Subsequently, the substrate was pulled off and tensile force versus displacement curves were recorded (see [6] for technical details).

**Figure 3:**
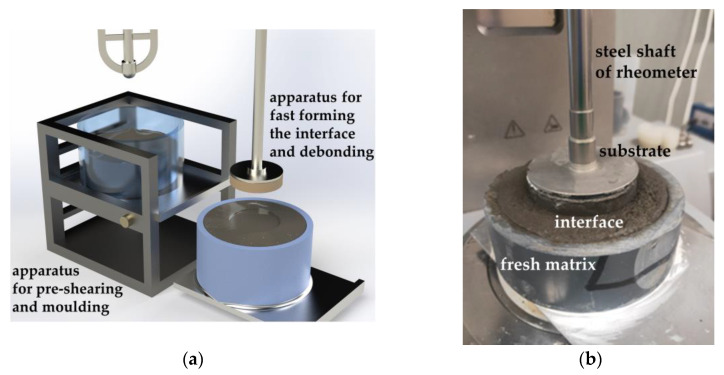
Fast tack test set-up: (**a**) sketch of the set-up for the fast tack test; (**b**) the formation of the interface in the fast tack test.

The modifications of the FTT were as follows: (1) A pre-shearing method was developed. (2) A method for fast formation of the interface between the substrate and fresh cementitious samples was established based on the pre-shearing. (3) The resting time was controlled to investigate the time-dependent interfacial adhesion. A sketch of the FTT is shown in Figure 3.

The size of the substrate was ∅ 50 mm×10 mm. The top surface of the substrate was adhered to a round metal plate (∅ 50 mm×1 mm) which can be directly connected with the steel shaft of the rheometer. The substrate was molded using UHPC. The root mean square of the bottom surface profile of the substrate was below 0.1 mm. The mix proportion of the UHPC is shown in Table 3.

**Figure 4:**
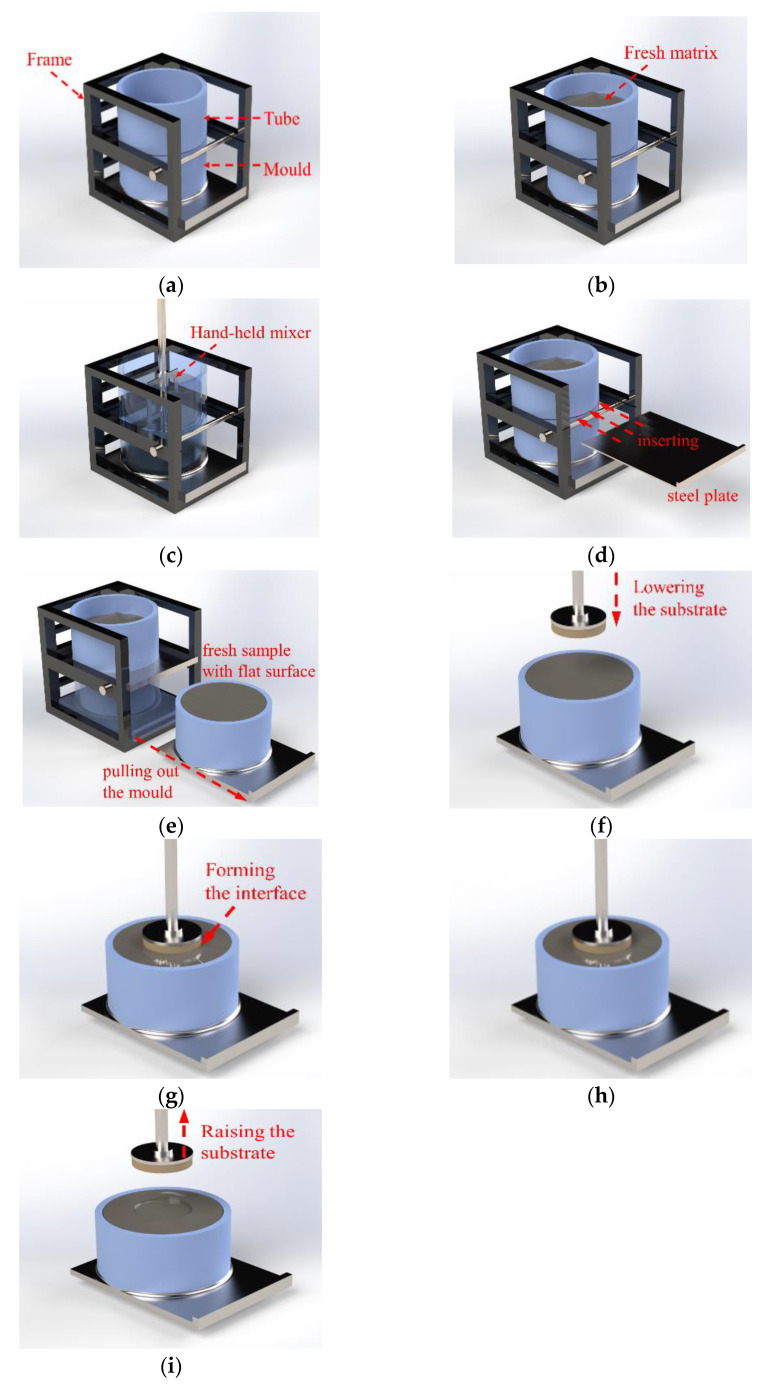
The testing process of the fast tack test: (**a**) the assembled apparatus; (**b**) adding matrix; (**c**) pre-shearing; (**d**) separating the mold; (**e**) pulling out the test sample; (**f**) lowering the substrate; (**g**) forming the interface; (**h**) resting; (**i**) debonding the interface.

The pre-shearing step aimed to break the flocculation of the fresh matrices. A pre-shearing and moulding apparatus was designed and used in the pre-shearing step. The apparatus consisted of a mold, a tube, and a frame, as shown in Figure 4a. The steel frame fixes the mold and the tube during pre-shearing. Positioning the tube on the mold concentrically creates a container for pre-shearing the matrices, as shown in Figure 4b. The mold had an inner diameter of 100 mm and a height of 30 mm. The tube had the same inner diameter as that of the mold and a height of 50 mm. The bottom of the mold was a flat steel plate on which the cylindrical part was glued. The fresh matrix was filled into the apparatus. The top surface of the fresh matrix needed to be above the mold and below the top of the tube. Then, a hand-held mixer was used to shear the matrix, as shown in Figure 4c. The bottom of the mixing paddle needed to sink into the mold.

The formation of the interface was highly punctual, with the precision of a second (unit). The fast formation of the interface consisted of 4 steps, as shown in Figure 4d–g. The fast formation of the interface took 15 s.

Once the pre-shearing finished, a steel plate was inserted into the joint between the mold and the tube, as shown in Figure 4d. With a sharp edge, the plate separated the matrix in the apparatus and formed a testing sample in the mold. Then the mold was pulled out from the frame and meanwhile formed the testing sample. The top surface of the sample was flat due to the flatness of the plate. The testing sample with the mold was placed directly on the testing plate of the rheometer. Double-sided tape was adhered on the testing plate of the rheometer in advance. Once the mold was placed on the testing plate, the tape bonded the mold to the testing plate. The substrate had been previously connected to the steel shaft of the rheometer. Once the sample was placed on the rheometer, the substrate was lowered by the displacement control mode of the rheometer. The substrate was pressed into the sample for 3 mm to form the interface, as shown in Figure 4e–g.

After the fast formation of the interface, the substrate remained still until debonding. The time from the moment when the substrate was in contact with the fresh sample surface until the moment of debonding was the resting time. The setting of the resting time aims to investigate the time-dependent evolution of the interface from the beginning of the interface formation. Debonding of the interface was performed in the displacement control mode. The debonding process is shown in Figure 4i.

The tensile stress is assumed to be uniformly distributed among the interface considering the equivalent displacement of each position in the substrate before the peak load. The peak value of the tensile force is recorded while lifting the substrate. The maximum tensile stress on the interface is calculated according to Equation (1) written as

(1) $$\sigma_{max}=\frac{F_{max}}{S}$$

where $\sigma_{max}$ represents the maximum tensile stress of the interface (Pa), *F_max_* represents the peak tensile force (N), and *S* represents the surface area of the substrate (m^2^).

In this study, 4 matrices were investigated (see Section 2.1 for details). For each matrix, 5 resting times were tested, i.e., 1, 4, 8, 16, and 32 min. The resting time was recorded from the moment when the substrate contacted the fresh matrix until the start of lifting the substrate. One sample was tested after each resting time. The test was conducted using an Anton Paar MCR 102 rheometer. The pre-shearing speed was 1200 rpm for 60 s. After the resting time, the lifting speed of the substrate amounted to 0.1 mm/s. The tensile force was recorded as a function of the imposed displacement. The displacement corresponding to the tensile force was recorded from the start of the lift.

#### 2.3.3. Fast Shear Tests

**Figure 5:**
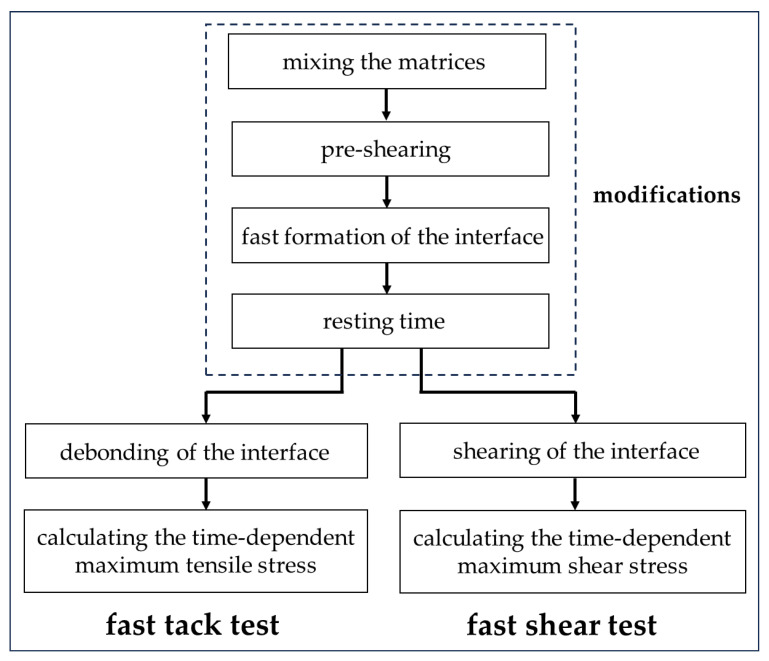
The scheme of the fast tack test and the fast shear test.

**Figure 6:**
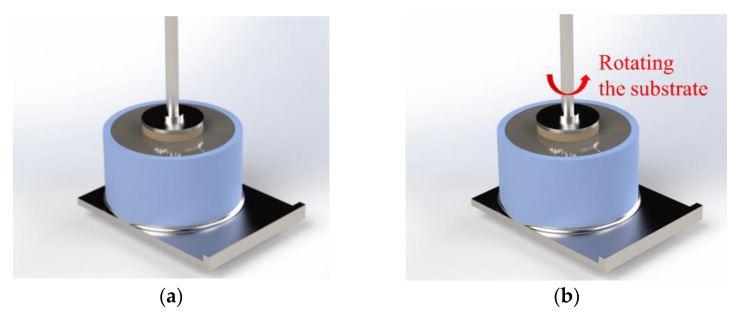
Shearing the interface in FST: (**a**) Resting; (**b**) Shearing the interface.

The fast shear test (FST) aims to measure the interface adhesion under shear stress between artificial rock and fresh cementitious materials. The test preparation and procedure of the FST was similar to the FTT. The substrate preparation, pre-shearing, and fast formation of the interface were identical to the FTT, which are detailed in Section 2.3.2. The difference between FST and FTT lies in the procedure of shearing the interface until the interface bond fails. When the resting time ended, the interface between the fresh matrix and the substrate in the FST was subjected to shear stresses. The shaft connected to the rheometer began to rotate at a fixed rotational speed. Meanwhile, the mold with the fresh sample was fixed by adhering the bottom of the mold to the rheometer. During the test, the vertical position of the substrate was kept the same. The scheme of the fast tack test and the fast shear test is shown in Figure 5. The shearing procedure of the interface is shown in Figure 6.

Based on elastic material constitutive relations, the maximum shear stress on the interface, which occurs at the perimeter, is calculated according to Equation (2) written as

(2) $$\tau_{max}=\frac{16T_{max}}{\pi d^{3}}$$

where $\tau_{max}$ represents the maximum shear stress of the interface (Pa), *T_max_* represents the peak torque value (N · m), *d* is the diameter of the substrate (m).

Four matrices were investigated (see Section 2.1 for details). For each matrix, 5 resting times were tested, i.e., 1, 4, 8, 16, and 32 min. One sample was tested after each resting time. The test was conducted using the Anton Paar MCR 102 rheometer. When forming the interface, the substrate was pressed into the top of the fresh sample for 3 mm. During shearing, the rotating speed of the substrate amounted to 0.1 rpm.

## 3. Results

### 3.1. Hydration Heat Release

**Figure 7:**
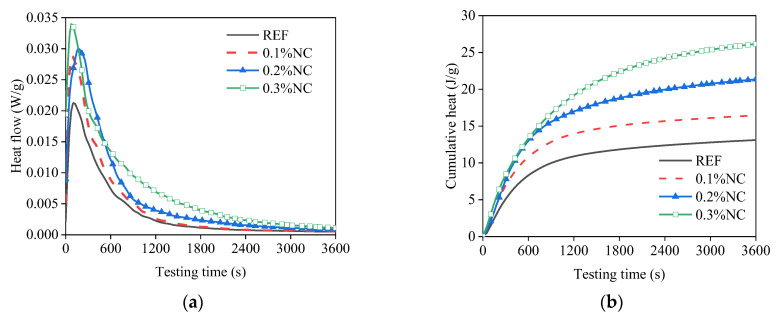
The hydration heat release of the matrices: (**a**) the heat release rate; (**b**) the cumulative heat release.

The addition of nanoclay increases the value of the first peak of hydration and the cumulative heat, as shown in Figure 7. When the addition of nanoclay increased from 0% to 0.3%, the peak value of the heat release rate increased by 61.9%, from 0.021 W/g to 0.034 W/g. The cumulative hydration heat increased by 99.2%, from 13.1 J/g to 26.1 J/g. The matrices with the addition of nanoclay showed extended heat release time in the first peak of hydration. The heat flow rate of the REF decreased to 0.001 W/g at around 2000 s. For the 0.3% NC, the decrease to 0.001 W/g occurred around 3600 s. The increased value of the heat flow and the extended heat release time resulted in a nearly doubled cumulative hydration heat in the first hour of hydration. Previous research indicates the nucleation effect of nanoclay can accelerate cement hydration [31].

### 3.2. Interfacial Adhesion under Tensile Stress

#### 3.2.1. The Tensile Force–Displacement Relations

**Figure 8:**
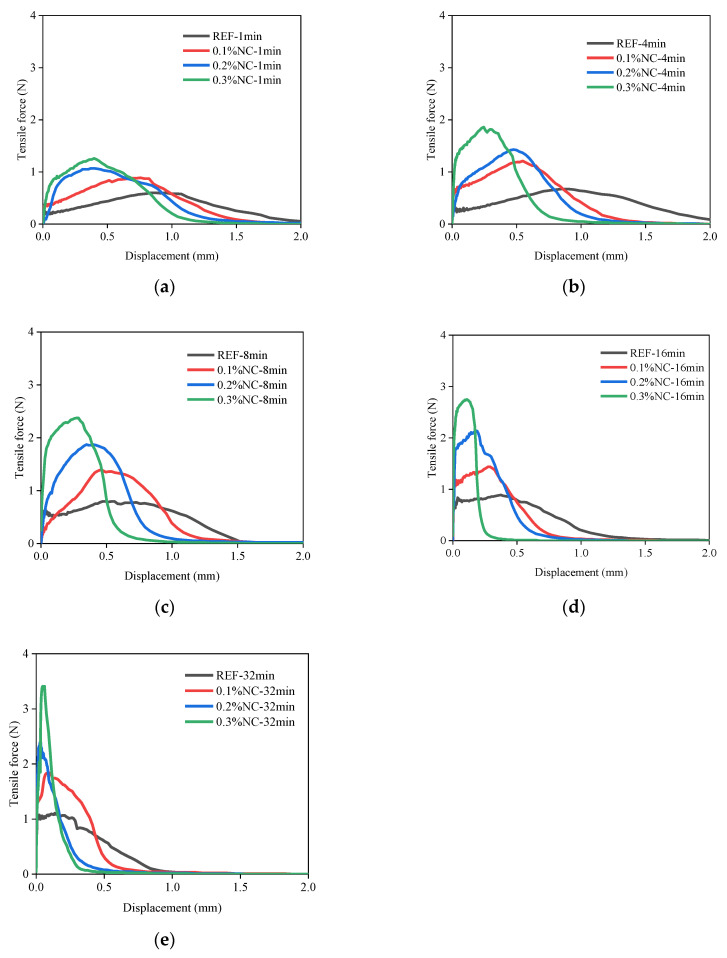
The tensile force–displacement relations with various resting times: (**a**) aesting time 1 min; (**b**) resting time 4 min; (**c**) resting time 8 min; (**d**) resting time 16 min; (**e**) resting time 32 min.

The tensile force–displacement relations after various resting times are shown in Figure 8. The maximum tensile force measured in the FTT is used to quantify the interfacial adhesion under tensile stress. The addition of nanoclay and the aging of matrices are two key factors influencing the tensile force–displacement relations. The interfacial adhesion increased step-wise as the dosage of nanoclay increased. Meanwhile, the interfacial adhesion increased with the aging of the matrices, as shown in Figure 8.

The shape of the tensile force–displacement curves evolved towards a more brittle failure pattern with the addition of nanoclay and the aging of the matrices. The curves changed from a smooth increase and decline to a much sharper increase and decline. The displacement corresponding to the maximum tensile force decreased as the nanoclay dosage increased and the matrices aged. The above-mentioned points can be observed when comparing the curves with a resting time of 1 min and those with a resting time of 32 min in Figure 8.

#### 3.2.2. The Interface Failure Modes

**Figure A1:**
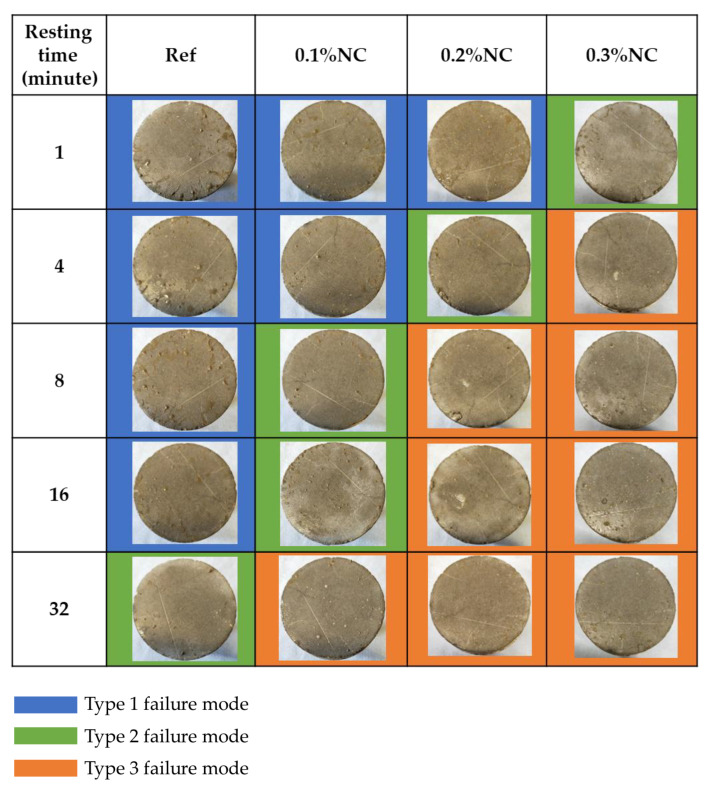
Interfacial tensile failure modes.

The debonding process was observed and the tensile failure modes of the interface are shown in Table 4. The photos and failure mode classification for each test sample are shown in Figure A1.

Debonding always occurred at the interface between the fresh matrix and the substrate. Three failure modes, labeled Type 1, Type 2, and Type 3, are characterized by the following features: Type 1 failure mode is characterized by the inward flow of the fresh matrices at the interface during the tensile process. The inward flow initiated from the fringe of the circular substrate and flew towards the center of the substrate, as shown in Table 4. With the flow, air penetrated the interface, and debonding developed from the fringe towards the center. Traces of fresh matrices caused by the inward flow can be observed on the substrate, as shown in the enhanced photo in Table 4. In Type 2 failure mode, the inward flow was also observed during the debonding process, but with a noticeably smaller air penetrating distance from the fringe towards the center. The decrease of the air penetrating distance from Type 1 to Type 2 was noticed in debonding, which is illustrated in Table 4. The displacement of the substrate corresponding to the complete debonding of the interface is smaller compared with Type 1. Small amounts of fresh matrices were left on the substrate in Type 2 failure mode. Different from Type 1, a few traces of fresh matrices were left on the fringe and more traces were distributed separately on the substrate. Type 3 failure mode showed more brittle characteristics compared with the others. No inward flow was observed during the debonding process. The displacement required for the complete debonding further decreased. The traces of the matrices left on the substrate were solid-like, in contrast to the fluid-like traces left in Type 1 and Type 2.

### 3.3. Interfacial Adhesion under Shear Stress

#### 3.3.1. The Torque–Rotational Angle Relations

**Figure 9:**
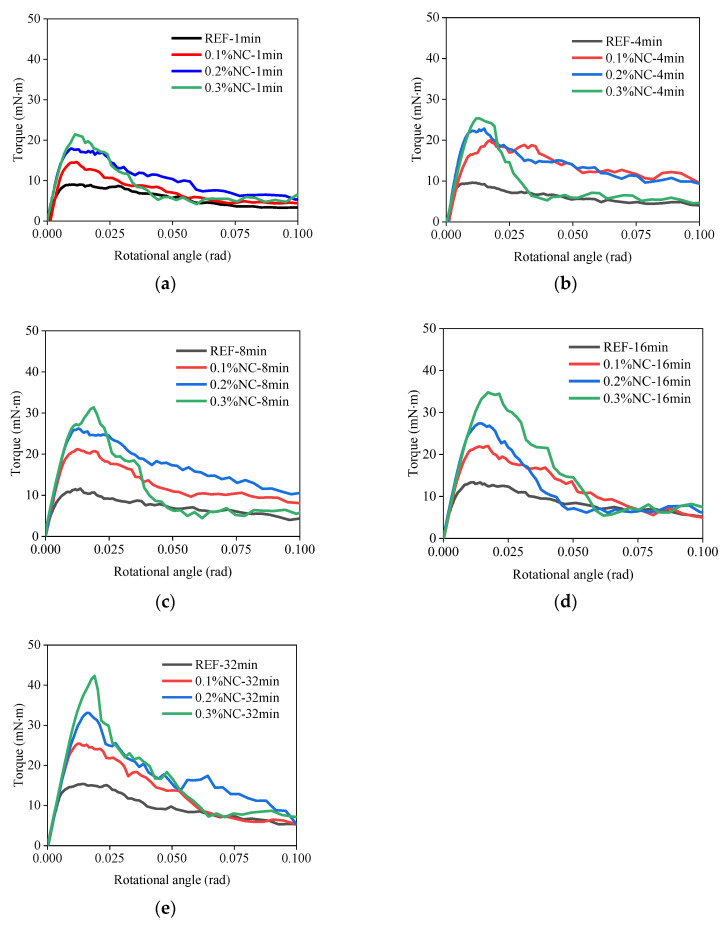
The torque–rotational angle relations with various resting times: (**a**) resting time 1 min; (**b**) resting time 4 min; (**c**) resting time 8 min; (**d**) resting time 16 min; (**e**) resting time 32 min.

The torque–rotational angle relations with various resting time are shown in Figure 9. The peak torque values measured in the FST ranged from 9.0 mN · m (sample REF-1 min) to 42.3 mN · m (sample 0.3%NC-32 min). The unit of mN · m was used based on the range of the peak torque values. The maximum torque of the interface was used to quantify the interfacial adhesion under shear stress. The addition of nanoclay and the aging of the matrices contributed to the interfacial adhesion. All test samples exhibited quasi-linear relations between torque and rotational angle up to the beginning of shearing. As the nanoclay dosage and the age of the matrix increased, the linear relation enhanced. Both the peak value of torque and the rotational angle corresponding to the peak value of the torque increased.

#### 3.3.2. The Interface Failure Modes

**Figure 10:**
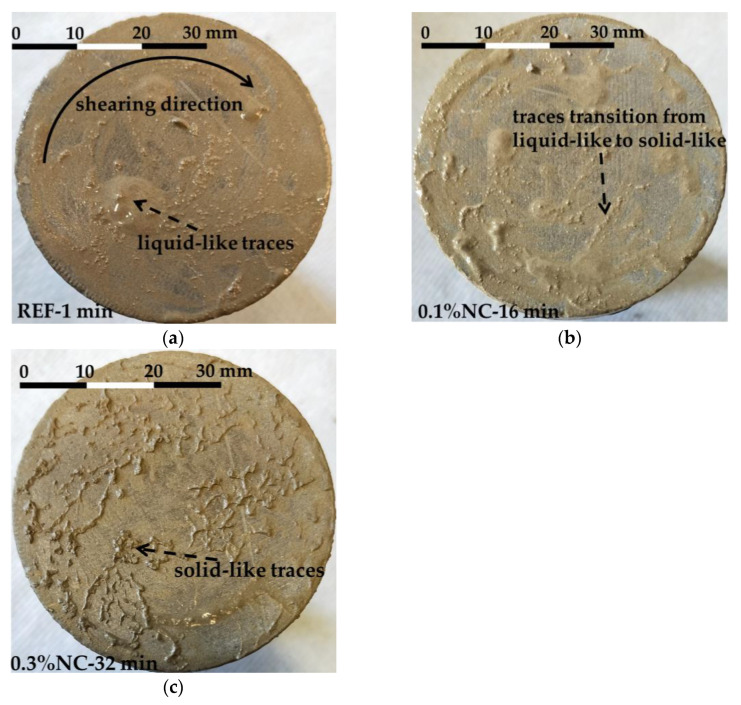
The failure modes of the interfaces subjected to shear stress: (**a**) Type 1 failure mode; (**b**) Type 2 failure mode; (**c**) Type 3 failure mode.

**Figure A2:**
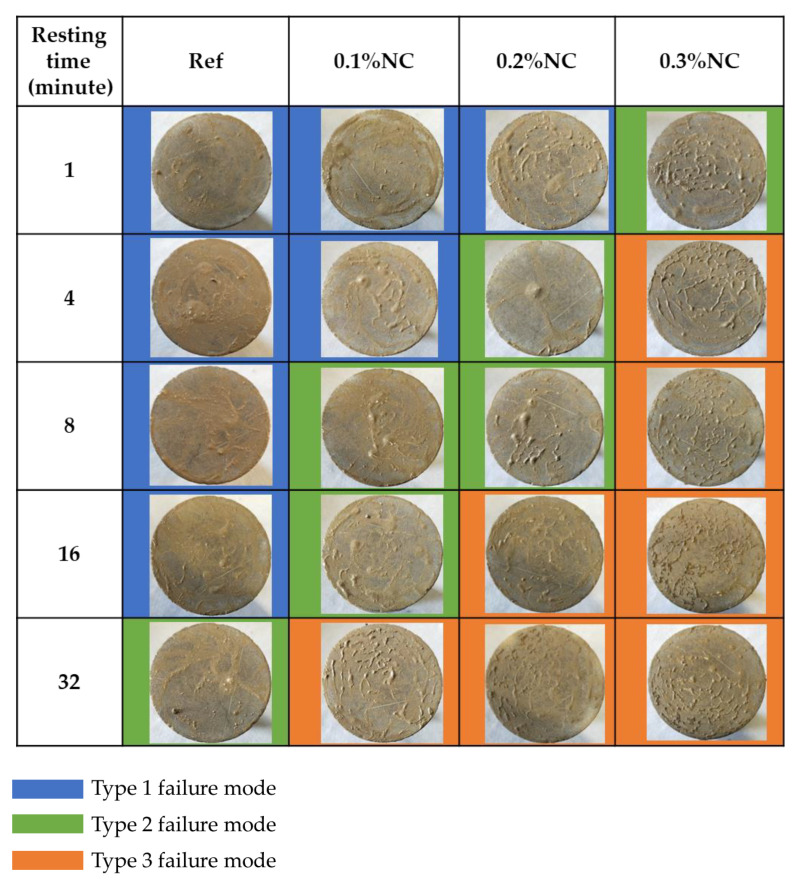
Interfacial shear failure modes.

The photos of the debonded substrate surfaces were recorded as shown in Figure A2. Three types of failure modes were observed and classified with the typical shear failure modes shown in Figure 10. The three failure modes were labelled as Type 1, Type 2, and Type 3. Type 1 shear failure mode was characterized by liquid-like traces left on the substrate. The traces appeared along the shearing direction. Type 2 failure mode was the transition state. The traces left on the substrate for Type 2 failure mode were stiffer when compared with Type 1. Type 3 failure mode showed solid-like traces after shearing, as shown in Figure 10.

## 4. Discussion

**Figure 11:**
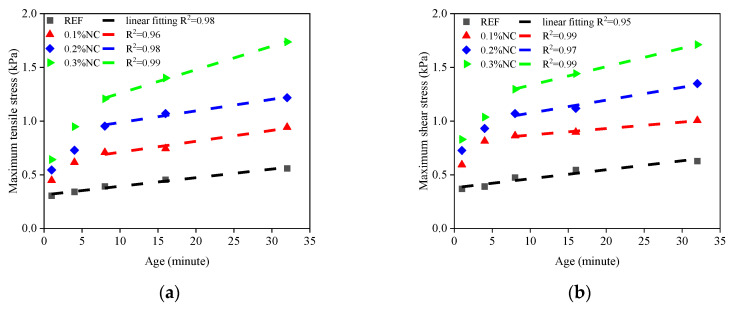
The time-dependent evolution of the interface adhesion: (**a**) the maximum tensile stress–age relations; (**b**) the maximum shear stress–age relations.

The test results show the effect of nanoclay on the time-dependent interfacial adhesion. The addition of nanoclay increases the maximum stress measured in the tests and alters the time-dependent evolution pattern of the maximum stress. The time-dependent maximum tensile stress and maximum shear stress are shown in Figure 11.

Firstly, increasing the nanoclay dosage in matrices consistently enhances the maximum tensile stress and maximum shear stress. This is evident throughout the entire test duration, i.e., from the 1st minute to the 32nd minute. The maximum tensile stress increases by 106%, from 0.31 kPa (REF) to 0.64 kPa (0.3% NC), when the resting time is 1 min. As the resting time increases, the samples modified by nanoclay show a faster increase in the interfacial adhesion. The maximum tensile stress increases by 209%, from 0.56 kPa (REF) to 1.73 kPa (0.3% NC), when the resting time is 32 min. The same trend is found in the maximum shear stress of the interface. The increase in the interfacial adhesion is higher than using viscosity modifying agents, e.g., redispersible polymer powder or cellulose ether [6]. The significant contribution of nanoclay to the interfacial adhesion shows the practical value of nanoclay in printable concrete. While previous research suggests that nanoclay can be used to enhance the buildability of printable concrete, the application scenario of the nanoclay is mainly confined to layer-by-layer deposited printing protocols [32]. This study proves the effect of an increase in nanoclay on the interfacial adhesion, which suggests that nanoclay can be used in further tunnel lining printing.

Meanwhile, the addition of nanoclay alters the evolution pattern of the maximum stress. The REF series exhibits a linear relationship in the time-dependent development of the maximum tensile stress and the maximum shear stress. With the addition of nanoclay, the increase of maximum adhesive stress shows two distinct stages. The first stage is characterized by a rapid stress increase, significantly outpacing the linear growth observed in the REF series. For instance, the increase in maximum tensile stress from the resting time of 1 min to 8 min is 0.09 kPa in the REF series, meanwhile, the increase is 0.56 kPa in the 0.3% NC series. The transition point occurs around the 8th minute, marking the shift from the first to the second stage. After this point, the increase rate for the 0.3% NC series is 0.022 kPa/min, while the increase rate for the REF series is 0.007 kPa/min. The second stage of the series modified by nanoclay mirrors the REF series with a linear increase in the maximum adhesive stress as the matrices age, albeit with a higher slope, due to the nanoclay presence. The two-stage increase fills the gap in understanding the time-dependent characteristics of the interfacial adhesion, especially at the fresh state, which was not discovered previously [7].

**Figure 12:**
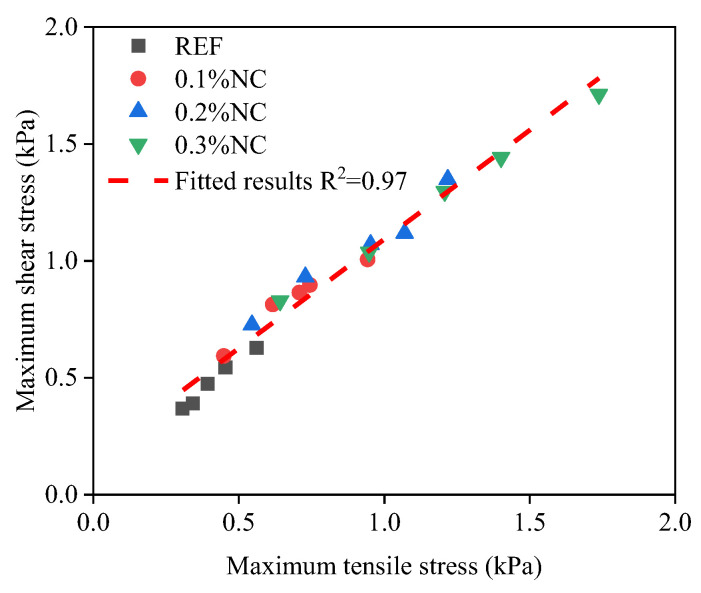
The relation between the maximum tensile stress and the maximum shear stress.

A linear relation is found between the interface adhesion in tension and shear, as shown in Figure 12. It indicates that the interfacial adhesion under tensile stress and the interfacial adhesion under shear stress keep nearly the same time-dependent development pattern.

The characteristics of each type of failure mode are explained in Section 3.2.2 for the FTT and Section 3.3.2 for the FST. In this section, the transition of the failure mode from Type 1 to Type 3 is discussed, including the mechanism behind each failure type and the driving factors contributing to the transition. The transitions of the interface failure modes for the FTT and the FST are discussed separately.

The transition of the interface failure modes in the FTT is illustrated in Figure A1. In Figure A1, the upper-left area is filled by the samples with Type 1 failure mode, while the lower-right area is filled by the samples with Type 3 failure mode. The diagonal area separating the upper-left area and the lower-right area is occupied by the samples with Type 2 failure mode. As shown in Figure A1, the dosage of nanoclay and the resting time of the samples are two key factors contributing to the transition of the failure modes. The increase of either factor contributes to the transition from Type 1 towards Type 3.

Type 1 failure mode is characterized by an inward flow and the penetration of air from the fringe of the substrate. During the interfacial debonding between a soft viscoelastic material (e.g., fresh cement paste) and a rigid substrate, the inward flow is normally observed when the test sample has a low resistance to flow [33]. For instance, Kawashima et al. [19] found that compared with the matrices with nanoclay, the plain cement paste showed gradual inward flow during the tack test. Mohamed et al. further pointed out that when inward flow appeared, the force required for separation was determined by the viscous properties of the soft material [34]. As the debonding process is accompanied by the inward flow, the whole process shows ductile features [19]. This is also observed in the FTT. The samples with Type 1 failure mode experienced larger displacement until debonding and the decline of the stress is smooth, as shown in Figure 8.

Type 2 failure mode represents a transferring mode from liquid-like debonding (Type 1) to a more solid-like debonding (Type 3). For characteristics of Type 2 failure mode, we refer to Section 3.2.2 for details. The stiffening of the fresh matrices is the main reason resulting in Type 2. Qian et al. [35] and Kawashima et al. [19] pointed out that the stiffening of the fresh matrices led directly to the change of interface failure mode. The factors resulting in the stiffening include the addition of nanoclay in the cementitious matrices [19] and the structural rebuilding of the matrices [35] over time. The two factors correspond to the horizontal and vertical variation within Figure A1 where samples with Type 2 failure mode are listed along the diagonal area.

Type 3 failure mode is shown in Figure 8 and Table 4. The further stiffening of the fresh matrices eliminates the inward flow. Meanwhile, the hydration of cement produces a chemical bonding on the interface. The solid-like particles adhered to the substrate indicate the increasingly important role of chemical bonding on the interface. A similar failure mode has been observed when debonding an adhesive from a rigid surface in tack tests [36]. In Type 3 failure mode, the fracture is accompanied by internal growth of voids, changing towards a brittle failure.

The transition of failure modes in the FST follows a similar trend as the above-mentioned transition in the FTT. The fresh matrices evolved from low-flow resistance (Type 1 failure mode) to a stiffening state (Type 2 failure mode) and further to forming chemical bonds on the interface (Type 3 failure mode). The detailed failure modes can be observed in Figure 10 and Figure A2. The addition of nanoclay and the aging of matrices contribute to the transition.

The addition of nanoclay has a stiffening effect on the cementitious matrices at the fresh state. When Type 1 failure mode appeared, the resistance to flow in the fresh matrices was directly connected with the interface adhesion. The inward flow needs to surpass the resistance of the fresh matrices while the addition of the nanoclay increases the flow resistance of the matrices.

The effect of nanoclay can be classified into two aspects. The first is the flocculation effect. Kawashima et al. [37] pointed out that the addition of nanoclay has an immediate stiffening effect on cementitious materials through flocculation. The immediate stiffening effect increases the flow resistance of fresh matrices from the beginning of the FTT and the FST, which results in the immediate increase in interfacial adhesion. The second aspect is that the addition of nanoclay increases the structural build-up rate of the microstructure of the matrices due to the nucleation effect [38]. Ma et al. [17] found the structural build-up of cementitious material consisted of two stages, namely, a faster non-linear increasing stage followed by a linear increasing stage. This corresponds with the time-dependent interfacial adhesion evolution previously discussed.

As the hydration proceeds, chemical bonds form on the interface between the fresh matrices and the substrate. In all FTT and FST, a thin layer of water was observed on the substrate surface right after the debonding of each sample, as shown in the appendices. While resting, the substrate and the fresh samples contacted each other (for details see Section 2.3). This provides physical connections between cementitious particles and the substrate surface in the presence of water. The hydration products formed in the vicinity of the interface and the bonding strengthened as the hydration proceeded.

The addition of nanoclay accelerates the hydration process of cementitious materials by increasing the number of nucleation sites for the growth of hydration products. For the fresh matrices, the hydration products formed during the Portland cement hydration comprise C-S-H gel, ettringite, calcium aluminate hydrates, and portlandite [39]. In this study, the effect of nanoclay on accelerating the hydration process was confirmed by the results of the isothermal calorimetry test. Both the peak value of the heat flow and the cumulative hydration heat increased with the addition of nanoclay. A similar accelerating effect of nanoclay on the hydration kinetics was reported by Wang et al. [31]. Considering the mixing procedure (for details see Section 2.2) and the formation of the interface (for details see Section 2.3.2 and Section 2.3.3), nanoclay is present and has a similar accelerating effect on the cementitious particles in the vicinity of the interface. With an increase in the dosage of nanoclay and the resting time, more hydration products formed on the interface, and this enhanced the interface bonding. As Rossol et al. [40] pointed out, the microstructural contact points turned gradually into far more rigid interactions as the hydration proceeded. The chemical bonding that gradually formed on the interface led to the time-dependent increase in the interfacial adhesion. Meanwhile, the increase in nanoclay dosage accelerated the hydration process, causing a higher interface adhesion after the same resting time.

## 5. Conclusions

This paper investigated the time-dependent interface adhesion between artificial rock and fresh mortar modified by nanoclay. With the fast tack test and the fast shear test, the effect of nanoclay on the interfacial adhesion was studied from the 1st minute until the 32nd minute after the formation of the interface. The following conclusions were obtained:

(1) The addition of nanoclay accelerates the hydration process of the fresh matrices. In the first hydration peak, the peak value of heat flow increases by 61.9% when the nanoclay dosage increases from 0% to 0.3% of the mass of the cement. Meanwhile, the accumulative hydration heat increases by 99.2%.
(2) The addition of nanoclay increases the interfacial adhesion. With nanoclay, the time-dependent interfacial adhesion first increases rapidly, then the increase slows down and follows a linear relation with the age of the matrix. There is a linear relation between the maximum tensile stress and the maximum shear stress. The addition of nanoclay instantly increases the resistance of the matrices to flow. This increases the force or torque needed to initiate the flow of matrices in the vicinity of the interface when debonding the interface. The nanoclay accelerates the formation of chemical bonds at the interface, which contributes to the interfacial adhesion.
(3) The addition of nanoclay contributes to the stiffening of the matrices. The stiffening effect accelerates the transition of the interface failure modes from a ductile failure mode towards a more brittle failure mode.
(4) The fast tack test and the fast shear test are useful for investigating the time-dependent interfacial adhesion between artificial rock and fresh mortar. The two tests can provide complete time-dependent development of the interfacial adhesion immediately after the premixing of the matrix.
(5) Nanoclay should be added to cementitious material to print concrete tunnel linings. The addition of nanoclay can enhance the time-dependent interfacial adhesion between rock and fresh mortar.

## Figures and Tables

**Table 1:** The mixture proportions of matrices (by the mass of cement).

Label	Cement	Sand	Water	Nanoclay
REF	1	1	0.35	0%
0.1%NC	1	1	0.35	0.1%
0.2%NC	1	1	0.35	0.2%
0.3%NC	1	1	0.35	0.3%

**Table 2:** Chemical composition and loss-on-ignition (LOI) of the Portland cement and the nanoclay (in % by weight).

	CaO	SiO_2_	Al_2_O_3_	Fe_2_O_3_	SO_3_	MgO	Na_2_O	Cl^−^	LOI	K_2_O	TiO_2_
Cement	64.1	18.6	5.2	4.9	3.5	1.2	0.6	0.1	1.8	-	-
Nanoclay	3.0	50.2	9.8	3.32	-	9.1	0.6	-	22.0	0.6	0.4

**Table 3:** The mixture proportions of the UHPC.

Materials	(kg/m^3^)
Sand 0/4	401.9
Basalt 4–8 mm	649.3
Silica Fume—Elkem Microsilica 940 U	153.8
Cement—Variodur 40	778.2
Filler—Betofill VK50 (limestone)	185.5
Water	186.4
Superplasticizer—SIKA Visconcrete UHPC-2	11.2

**Table 4:** The tensile failure modes of the interface.

Failure Mode	Illustration of the Debonding	Interface Failure Mode after the Debonding
Type 1	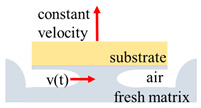	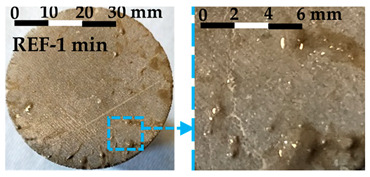
Type 2	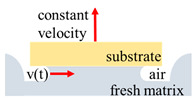	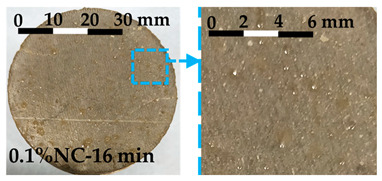
Type 3	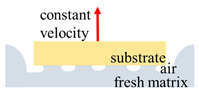	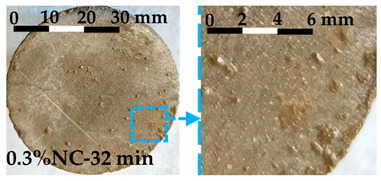

## Data Availability

The data presented in this study are available from the corresponding author upon request.
